# Tooth wear among five-year-old children in Jakarta, Indonesia

**DOI:** 10.1186/s12903-019-0883-5

**Published:** 2019-08-20

**Authors:** Diah Ayu Maharani, Alisa Novianty Pratiwi, Febriana Setiawati, Shinan Zhang, Sherry Shiqian Gao, Chun Hung Chu, Anton Rahardjo

**Affiliations:** 1 Department of Preventive and Public Health Dentistry, Faculty of Dentistry, Universitas Indonesia, Jalan Salemba No. 4, Jakarta, 10430 Indonesia; 2 School of Stomatology, Kunming Medical University, Yunnan, China; 3 Faculty of Dentistry, The University of Hong Kong, Hong Kong, China

**Keywords:** Tooth wear, Children, Oral health behaviors, Epidemiology

## Abstract

**Background:**

This study aimed to evaluate the prevalence of tooth wear among preschool children in Jakarta, Indonesia, and examine the risk factors associated with its occurrence.

**Methods:**

An epidemiological survey was conducted with a cross-sectional study design. The participants were recruited via cluster sampling. Tooth wear was clinically assessed by one examiner using the Basic Erosive Wear Examination (BEWE) criteria. The children’s caries experience was also recorded. The parents of the participating children completed a self-administered questionnaire to answer demographic questions about the children and gather information about the children’s diet and oral health behaviors as well as the parents’ dental health-related knowledge. The data were analyzed using the Chi-square test and binary logistic regression.

**Results:**

A total of 752 five-year-old children were invited to participate, with 691 (92%) enrolling in the study. Tooth wear occurred in 23% (161/691, BEWE > 0) of the participants, in which 78% (125/161) had at least one moderate tooth wear status (BEWE = 2). The consumption of citrus drinks, fruit juice, and vitamin C supplement drinks, together with the child’s caries experience, the father’s education level, and the family’s socioeconomic status, were significantly associated with tooth wear.

**Conclusions:**

The five-year-old preschool children in Jakarta had a relatively low prevalence of tooth wear. Those consuming more acidic drinks, those with a higher socioeconomic status, and those with an absence of caries experience had a higher risk of tooth wear.

**Electronic supplementary material:**

The online version of this article (10.1186/s12903-019-0883-5) contains supplementary material, which is available to authorized users.

## Background

Tooth wear is described as the irreversible loss of dental hard tissue due to the chemical influence of extrinsic acid (including acid from diet and medications) and intrinsic acid (including acid from gastroesophageal reflux and vomiting) without bacterial involvement [1, 2]. This leads to the loss of the chemically softened oral tissue by abrasive forces [3]. It is a multifactorial condition involving an interplay of various chemical, biological, and behavioral factors. The potential for tooth wear depends on chemical factors including pH, titratable acidity, mineral content, and the calcium-chelating properties of the dental tissue. Biological factors, such as saliva, acquired pellicle, tooth structure, and tooth position in relation to the soft tissues and the tongue, are correlated with the pathogenesis of tooth wear. Furthermore, behavioral factors, including eating and drinking habits, and excessive oral hygiene are predisposing factors for tooth wear [4].

The prevalence of tooth wear varies widely worldwide, suggesting the effect of the diverse diet habit among different countries or regions on tooth wear. An epidemiological survey performed in Greece reported that 98.4%, of preschool children presented with tooth wear [5]. Similarly, tooth wear was present in 75% of Australian preschool children [6]. Moreover, roughly half (45%) of preschool children in Germany had tooth wear [7]. These results improved among Asian children. Tooth wear was prevalent in 18% of five-year-old children in Hong Kong [8], while in China, the prevalence of tooth wear was reported in 15% of children in Shanghai and only 6% in Guangxi and Hubei [9, 10]. Although the prevalence of tooth wear was found to be relatively low in several Asian places compared to Western countries, limited information is available regarding the situation in Indonesia.

Primary tooth enamel is thinner and softer compared to that of permanent teeth, and morphological differences exist between primary and permanent teeth. Therefore, the process of tooth wear can be faster in primary teeth. Even short-term exposure to acids may lead to advanced lesions progressing to the dentine in primary teeth [2]. Tooth wear may lead to tooth sensitivity, altered occlusion, compromised aesthetics, and, in severe cases, pulp exposure [1]. Moreover, the presence of early tooth wear damage to primary teeth can compromise dentition for the child’s entire lifetime. Tooth wear in primary dentition is considered a predictor of the increased risk of tooth wear in permanent dentition [10].

The dental treatment of tooth wear can be complex, expensive, and challenging; thus, early diagnosis is important. An epidemiological examination of young children with the tooth wear-induced loss of tooth structure should be performed to monitor the prevalence and severity, and preventive measures should be planned and provided as early as possible. The World Health Organization recommends the reporting of preschool children’s oral health status starting at the age of five. However, data regarding the prevalence of tooth wear among five-year-old preschoolers in Indonesia are scarce. Therefore, the aims of the present study were to evaluate the prevalence of tooth wear in five-year-old preschoolers in Jakarta, Indonesia, and identify the risk factors for tooth wear in this patient population.

## Methods

This study was conducted in Jakarta, Indonesia, between January and May 2017. The reporting of the present study is in accordance with the Strengthening the Reporting of Observational Studies in Epidemiology (STROBE) statement [11].

### Sample size estimation and selection of children

No study has reported on the prevalence of tooth wear in deciduous teeth in Indonesia. The prevalence of tooth wear varies widely between countries. There is high heterogeneity between studies, which is influenced by methodological and diagnosis factors [12]. For the purposes of this study, we estimated that tooth wear occurs in 20% of the population. The margin of error for the estimate was set at 3%. With a two-sided 95% confidence interval (CI), the required sample size was 683 children. With an estimated response rate of 90%, a total of 758 children aged 5 years were invited to join this study. A cluster sampling method was used to recruit the participants. Jakarta has six districts. The number of kindergartens chosen in each district was based on the proportion of the population living in the district. The government provided a list of kindergartens, which were randomly selected by personal computer for each district for inclusion in the study. All five-year-old children attending the selected kindergartens were invited to take part in the study. Written informed consent was provided by the parents of the participating children.

### Questionnaire survey

The parents or guardians of each child were asked to complete an onsite and self-administered questionnaire that was adapted from questions used in a previous study [13] (attached as Additional file 1). The questionnaire included demographic information (sex of the child, education level of the parents, and socioeconomic status of the family), information about the child’s oral health–related behaviors (intake frequency of soft drinks, citrus drinks, fruit juice, and vitamin C supplement drinks, as well as usage of chewing gum, tooth-brushing practices, and history of dental visits), information about the presence of digestive disorders in the child, and information about the parents’ dental knowledge. There were 21 multiple-choice questions regarding the causes and prevention of dental diseases in order to assess the parents’ dental knowledge. Each correct answer was allotted one point, and no score was given for an incorrect or “I don’t know” answer. Thus, the total dental knowledge score ranged from 0 to 21. Based on the total scores, the level of knowledge was then categorized into three groups using three equal intervals: low (0–7), moderate (8–14), and high (15–21).

### Clinical examination

A trained and calibrated dentist performed all the clinical examinations using a ball-end Community Periodontal Index probe and a disposable dental mirror attached to an intraoral light-emitting diode light. Duplicate examinations were conducted in 10% of the children at each school visit. A kappa statistic was used to test intra-examiner reproducibility. Tooth wear status was evaluated using the Basic Erosive Wear Examination (BEWE) criteria [14], which were used to examine the buccal, lingual, and occlusal/incisal surfaces of all primary teeth. The severity of tooth wear was recorded at 4 levels: 1) score of 0 = no tooth wear; 2) score of 1 = initial loss of surface texture; 3) score of 2 = distinct defect with hard tissue loss of less than 50% of the surface area (often involving the dentin); and 4) score of 3 = hard tissue loss of greater than 50% of the surface area (often involving the dentin). The surface with the highest BEWE score in a sextant was recorded to represent the entire sextant. Because of its sensitivity and specificity, the BEWE does not affect the diagnosis of dental caries. In addition, after the BEWE examination was conducted, we used the Decayed, Missing, and Filled Teeth (DMF-T) index to evaluate the dental caries experience of the primary teeth, which was assessed according to the World Health Organization criteria [15].

### Statistical analysis

Two investigators entered the data from the clinical examinations and questionnaires into an Excel spreadsheet. Data cleaning was performed before the analysis. A descriptive analysis was performed to establish the prevalence of tooth wear among the participants. The children were divided into two groups: those with tooth wear (BEWE score > 0) and those without tooth wear (BEWE score = 0). The relationships between the presence of tooth wear and all the independent variables (demographic information, the child’s oral health–related behaviors, the presence of a digestive disorder in the child, and the parents’ dental knowledge) were assessed using Chi-square analysis. Next, all the variables were evaluated using a logistic regression model to determine the risk factors for tooth wear in the participants. The variables that were not statistically significant were removed via backward stepwise regression. The final model only included statistically significant variables. The level of statistical significance for all the tests was set at 0.05.

## Results

Overall, 752 children from 33 kindergartens were invited to participate in the study; of these, 61 children were excluded because they did not provide a complete consent form or questionnaire or did not receive an oral examination due to uncooperative behavior. Accordingly, 691 children (370 boys and 321 girls) were included in the present study (a 91% response rate). Among the participants, 195 were in East Jakarta, 61 were in Central Jakarta, 157 were in West Jakarta, 147 were in South Jakarta, 113 were in North Jakarta, and 18 were in the Thousand Islands. The kappa value for the evaluation of tooth wear was 0.94. The prevalence of DMF-T was 82.5%, with a mean index of 7.20 (SD = 5.94).

### Tooth wear status

In total, 161 children (95 boys and 66 girls) were found to have at least one tooth wear lesion. In the present study, the prevalence of tooth wear was 23%, with a mean index of 0.29 (SD = 0.61). Among the children with tooth wear, the majority (78%) presented with a distinct defect consisting of hard tissue loss of less than 50% of the surface area (BEWE score = 2), while none of the children had severe tooth wear (BEWE score = 3) (Table 1). The mandibular anterior sextant showed the highest prevalence of tooth wear (19%), with a statistically significant difference compared to other sextants. This was followed by the maxillary anterior sextant (6%) (Table 2). Tooth wear was not prevalent in the posterior teeth of the young children.

**Table 1:** Prevalence and severity of tooth wear in 5-year-old children

	No.	BEWE > 0 No. (prevalence)	Highest BEWE Score No. (prevalence)			
			0	1	2	3
Sex						
Male	370	95 (26%)	275 (74%)	20 (5%)	75 (20%)	–
Female	321	66 (21%)	255 (79%)	16 (5%)	50 (16%)	–
Total	691	161 (23%)	530 (77%)	36 (5%)	125 (18%)	–

**Table 2:** Distribution of the severity of tooth wear in each sextant

Sextant	BEWE Score No. (prevalence)			
	0 (no erosion)	1 (initial loss)	2 (hard tissue loss < 50%)	3 (hard tissue loss > 50%)
Upper anterior teeth	648 (93.8%)	11 (1.6%)	32 (4.6%)	–
Upper right posterior teeth	680 (98.4%)	7 (1.0%)	4 (0.6%)	–
Upper left posterior teeth	686 (99.3%)	1 (0.1%)	4 (0.6%)	–
Lower anterior teeth	559 (80.9%)	27 (3.9%)	105 (15.2%)	–
Lower right posterior teeth	667 (99.4%)	2 (0.3%)	2 (0.3%)	–
Lower left posterior teeth	685 (99.1%)	2 (0.3%)	4 (0.6%)	–

### Risk factors related to tooth wear

The results of the Chi-square test showed that the intake frequency of citrus drinks, fruit juice, and vitamin C supplement drinks, in addition to caries experience, were significantly related to the incidence of tooth wear (Table 3). The results of the logistic regression analysis are provided in Table 4. The final model obtained by backward stepwise binary regression was the same as that obtained from the forward stepwise procedure. The children with a higher intake frequency of citrus drinks (OR = 2.41, *p* < 0.001), fruit juice (OR = 2.01, *p* = 0.001), and vitamin C supplement drinks (OR = 2.21, *p* = 0.017) had a higher incidence of tooth wear. Moreover, the children from high socioeconomic backgrounds (OR = 1.66, *p* = 0.012) or with fathers with a lower education level had a high risk of tooth wear. In addition, the children with caries experience had a lower incidence of tooth wear (OR = 0.52, *p* = 0.004).

**Table 3:** Tooth wear according to the studied variables

Variables	N	BEWE > 0 No. (prevalence)	*p*-value
Children examined	691	161 (23%)	
Sex			
Male	370	95 (26%)	0.113
Female	321	66 (21%)	
Frequency of soft drink intake			
Less than once every 2 days	679	156 (23%)	0.129
At least once every 2 days	12	5 (42%)	
Frequency of citrus drink intake			
Less than once every 2 days	590	119 (20%)	< 0.001
At least once every 2 days	101	42 (42%)	
Frequency of fruit juice intake			
Less than once every 2 days	527	105 (20%)	< 0.001
At least once every 2 days	164	56 (34%)	
Frequency of vitamin C supplement drink intake			
Less than once every 2 days	641	138 (22%)	< 0.001
At least once every 2 days	50	23 (46%)	
Frequency of chewing gum consumption			
Less than once every 2 days	34	12 (35%)	0.090
At least once every 2 days	657	149 (23%)	
Methodology used for drinking			
Drinking with straw and swallowing immediately	582	131 (23%)	0.524
Drinking from glass and swallowing immediately	98	27 (28%)	
Swishing before swallowing	11	3 (27%)	
Frequency of daily toothbrushing			
≥ 2 times	549	126 (23%)	0.670
≤ 1 time	142	35 (25%)	
Socioeconomic status			
Low	348	72 (21%)	0.102
High	343	89 (26%)	
Father’s education level			
College or above	407	82 (20%)	0.064
Secondary school	244	68 (28%)	
Primary school or below	40	11 (28%)	
Mother’s education level			
College or above	370	78 (21%)	0.129
Secondary school	270	66 (24%)	
Primary school or below	51	17 (33%)	
Parent’s dental knowledge			
High	206	43 (21%)	0.374
Moderate	435	103 (24%)	
Low	50	15 (30%)	
Presence of digestive disorders			
No	605	140 (23%)	0.793
Yes	86	21 (24%)	
Dental visit within the last 12 months			
Yes	221	48 (22%)	0.500
No	470	113 (24%)	
Caries experience			
No	121	40 (33%)	0.007
Yes	570	121 (21%)

**Table 4:** Logistic regression analyses of odds for tooth wear (BEWE > 0) among Indonesian children

Variables	OR	95% CI	p-value
Frequency of citrus drink intake			
Less than once every 2 days^reference^			
At least once every 2 days	2.41	1.48–3.93	< 0.001
Frequency of fruit juice intake			
Less than once every 2 days^reference^			
At least once every 2 days	2.01	1.34–3.03	0.001
Frequency of vitamin C supplement drink intake			
Less than once every 2 days^reference^			
At least once every 2 days	2.21	1.15–4.26	0.017
Father’s education level			
College or above^reference^			
Secondary school	1.83	1.21–2.76	0.004
Primary school or below	2.07	0.93–4.60	0.075
Socioeconomic status			
Low^reference^			
High	1.66	1.12–2.48	0.012
Caries experience			
No^reference^			
Yes	0.52	0.33–0.81	0.004

*OR*odds ratio, *CI* confidence interval, *reference* reference group; R^2^ = 0.121; *p*-values were calculated using binary logistic regression analyses

## Discussion

Approximately 600 million people live in Southeast Asia, which comprises 9% of the world’s population. As the fourth most populated country in the world, Indonesia has the largest population in Southeast Asia [16]. Surprisingly, in spite of its large population, a search revealed that no epidemiological study has reported on the oral health status of five-year-old children in Indonesia [17]. The present study is the first oral health survey of Indonesian preschool children to report on the tooth wear status of primary teeth.

Various diagnostic criteria are readily available for the evaluation of tooth wear, including the BEWE criteria [14], the Smith and Knight tooth wear index [18], and the simplified scoring criteria for tooth wear index [19]. However, there is no consensus regarding a standardized instrument for use in epidemiological surveys. Introduced in 2007, the BEWE index is used to identify tooth wear at the patient level, and this has been adopted in epidemiological surveys worldwide [13, 17]. In the present study, we assessed tooth wear using the BEWE index because it is a validated and simple instrument that is less time-consuming, and more practical compared to other measures, especially when used in young children. In addition, the use of a widely adopted instrument allows our results to be compared with findings from other places, which increases the impact of the present study.

The survey results demonstrated a 23% prevalence of tooth wear in Jakarta preschool children, which was much lower than that of children from Western countries (98.4, 75, and 45.4% in Greece, Australia, and Germany, respectively [5–7]). However, the prevalence was slightly higher than that of Chinese preschoolers, which was reported by previous studies to be 14.9, 5.7, and 15.1% [8–10]. The prevalence of tooth wear in the anterior teeth was higher than in the posterior teeth, which is consistent with results from other studies [8, 20]. This may be because early eruption, together with the location of the anterior teeth, can result in a longer contact time between acidic drinks and the anterior teeth when compared with the posterior teeth.

In the studied population, the higher intake frequency of citrus drinks, fruit juice, and vitamin C supplement drinks was related to a higher likelihood of tooth wear, which is consistent with previous reports [21, 22] suggesting a dose–response relationship between the consumption of acidic drinks and tooth wear. One study reported that not only the intake frequency of acidic drinks but also the length of time taken to consume these drinks were risk factors for tooth wear [23], as the increased contact time between acidic beverages and teeth can result in a pronounced and prolonged drop in the pH value of the oral cavity. Some studies suggested that tooth wear was associated with drinking methods, a finding that is considered to be related to the contact time [24]. The drinking method was not a risk factor for tooth wear in the present study. However, only one question applied to the methods used for drinking, which may not have provided sufficient information to assess the contact time between acidic drinks and teeth. Follow-up questions regarding the details of the children’s drinking behaviors should be developed to collect more information.

This study identified a significant relationship between high socioeconomic status and the prevalence of tooth wear, in accordance with another study exhibiting similar results [20]. Although it has been debated whether families with a high socioeconomic status typically have healthier dietary habits, such habits increase their children’s chances of consuming more acidic drinks, such as the citrus drinks and fruit juice that we identified as risk factors for tooth wear in this study. Furthermore, the different dietary habits of various countries and regions should also be carefully considered, as diet can influence the occurrence of tooth wear [25]. Conversely, the children whose fathers had a lower education level had a higher risk of tooth wear. In Indonesia, the male is the dominant member of the family. Perhaps the fathers’ lower education level may have led to unhealthy dietary habits in the children with tooth wear; however, this connection was not straightforward, and the results conflicted with those reported by other studies [8, 20]. This cross-sectional study has limitations in that it may not be possible to find or verify causal relationships between various factors. It must be taken into consideration that tooth wear is a cumulative effect of multifactorial processes, which warrants the need for long-term future studies.

Some studies either did not identify a significant relationship between the incidence of tooth wear and dental caries [26] or found a simultaneous relationship [8]. In the present study, the children with dental caries had a lower risk of tooth wear. Our findings were similar to those of a previous study that observed an inverse relationship between the incidence of tooth wear and dental caries [27]. However, previous findings from published data are equivocal, with some studies reporting a statistically significant association between tooth wear and dental caries [28, 29], while others do not [30, 31]. Since the number of caries-free children was small in the present study, the results observed here should be interpreted with caution. More studies are, therefore, needed to clarify this association.

Our study has some limitations, which must be addressed. First, the study findings were restricted to children in Jakarta, random errors due to the cluster sampling may have occurred, and the results may not be generalized to all children in Indonesia. Future studies should be performed in other populations in Indonesia and also with different age groups, as conducted in other countries, to gain a better understanding of tooth wear [32]. A longitudinal study should be carried out in the future for better elucidation of the risk factors for tooth wear [33]. Second, it would have been ideal to provide a reexamination for duplicates after 1 week; however, this was not possible because the kindergartens refused a second visit.

Conversely, this study has several significant strengths. First, a large sample size (691 children) was used. Moreover, a high response rate (91%) was achieved by cluster sampling, and intra-examiner agreement was very high (0.94). Second, this study is the first report on tooth wear in Indonesian preschool children and helps to fill an information gap in the literature. This study identified a lower prevalence of tooth wear in Indonesian compared to European preschool children [5, 7]. However, the Indonesian children had a higher prevalence of tooth wear compared to those in other Asian countries [8–10]. Tooth wear correlated with the children’s dietary behaviors; therefore, educational programs designed to decrease the consumption of acidic drinks should be adopted. In addition, this study showed that children from high socioeconomic backgrounds demonstrated a higher risk of tooth wear. Hence, strategies or programs to treat tooth wear should be prioritized in such high-risk children. Furthermore there is evidence that in Indonesia there have been dietary changes in the direction of Western food preferences, which may lead to risks for tooth wear [34]. As the world’s fourth most populous country, the burden of such disease may cause significant negative impacts.

## Conclusion

The prevalence of tooth wear in five-year-old Jakarta preschoolers was relatively low. The findings suggest that higher risk of tooth wear may be associated with the following elements: a higher intake of citrus drinks, fruit juice, and vitamin C supplement drinks; the father’s lower education level; a higher socioeconomic background; and absence of dental caries.

## Additional file

Additional file 1: Oral health questionnaire. (PDF 210 kb)

## Data Availability

The raw data is available from the authors for any author who wishes to collaborate with us.

## Abbreviations

BEWE: Basic Erosive Wear Examination
CI: Confidence Interval
OR: Odds Ratio
