# Supplementary material for: Tooth wear among five-year-old children in Jakarta, Indonesia
Source: BMC Oral Health. 2019 Aug 20;19:192. doi: 10.1186/s12903-019-0883-5 (PMC6702728; doi:10.1186/s12903-019-0883-5)
Supplement: Supplementary file 1 — Oral health questionnaire. (PDF 210 kb) [file 12903_2019_883_MOESM1_ESM.pdf]

Oral Health Questionnaire

Child name :

Gender : □Male □Female

Place of birth :

Date of birth (DD-MM-YYYY):

Class :

School :

Family monthly income:

Contact number (for further follow-up):

The educational level of the parents who live with the child:

	Father	Mother
Primary or below	□	□
Secondary	□	□
College or above	□	□

How often does your child brush his/her teeth?

□ Never / irregularly

□ Once a day

□ Twice a day

□ Three times a day or more

Did your child visit a dentist in the last 12 months?

□ Yes □ No

Does your child have digestive disorder or vomits frequently?

□ Yes □ No

Part A: Dietary habit of your child

1) How often does your child have soft drinks?
   - □ 6, 7 times a week or more (almost every day or more)
   - □ 3 times a week (alternate days)
   - □ Once a week
   - □ Less than once a week / never

2) How often does your child have citric tea / drinks containing lemon?
   - □ 6, 7 times a week or more (almost every day or more)
   - □ 3 times a week (alternate days)
   - □ Once a week
   - □ Less than once a week / never

3) How often does your child drink fruit juice?
   - □ 6, 7 times a week or more (almost every day or more)
   - □ 3 times a week (alternate days)
   - □ Once a week
   - □ Less than once a week / never

4) How often does your child have vitamin C supplement drinks?
   - □ 6, 7 times a week or more (almost every day or more)
   - □ 3 times a week (alternate days)
   - □ Once a week
   - □ Less than once a week / never

5) How often does your child have chewing gum?
   - □ 6, 7 times a week or more (almost every day or more)
   - □ 3 times a week (alternate days)
   - □ Once a week
   - □ Less than once a week / never

Pert B: Parent’s dental knowledge

1) The causes of dental decay include:

	Yes	No	Don’t know
a) Too much consumption of candies	◻	◻	◻
b) Unclean teeth	◻	◻	◻
c) Tooth worms attack	◻	◻	◻
d) “Hot air”	◻	◻	◻

2) Preventions of tooth decay include:

	Yes	No	Don’t know
a) Using miswak	◻	◻	◻
b) Gargle with salted water	◻	◻	◻
c) Use of fluoridated toothpaste	◻	◻	◻
d) Decrease frequency of sugar consumption	◻	◻	◻

3) Effects of fluoride to teeth include:

	Yes	No	Don’t know
a) No effect	◻	◻	◻
b) Prevent tooth decay	◻	◻	◻
c) Tooth whitening	◻	◻	◻
d) Prevent periodontal disease	◻	◻	◻

4) Which of the following food can cause tooth decay?

	Yes	No	Don’t know
a) Soft drinks	◻	◻	◻
b) Ice-cream	◻	◻	◻
c) Cheese	◻	◻	◻
d) Peanuts	◻	◻	◻

5) The causes of gum bleeding include:

	Yes	No	Don’t know
a) Unclean teeth	◻	◻	◻
b) It Is a normal phenomenon	◻	◻	◻

6) Methods to prevent periodontal disease include:

	Yes	No	Don’t know
a) Tooth brushing	◻	◻	◻
b) Saline mouth-rinsing	◻	◻	◻
c) Regular scaling (professional tooth cleaning)	◻	◻	◻
